# Effect of the Food Additives Sodium Citrate and Disodium Phosphate on Shiga Toxin-Producing *Escherichia coli* and Production of *stx*-Phages and Shiga toxin

**DOI:** 10.3389/fmicb.2016.00992

**Published:** 2016-06-23

**Authors:** Lucas J. Lenzi, Paula M. A. Lucchesi, Lucía Medico, Julia Burgán, Alejandra Krüger

**Affiliations:** ^1^Laboratorio de Inmunoquímica y Biotecnología, Facultad de Ciencias Veterinarias, Universidad Nacional del Centro de la Provincia de Buenos Aires, TandilArgentina; ^2^Centro de Investigación Veterinaria de Tandil, CONICET-CIC-UNCPBA, TandilArgentina

**Keywords:** STEC, Shiga toxin, bacteriophages, food additives, sodium citrate, disodium phosphate

## Abstract

Induction and propagation of bacteriophages along the food production chain can represent a significant risk when bacteriophages carry genes for potent toxins. The aim of this study was to evaluate the effect of different compounds used in the food industry on the growth of Shiga toxin-producing *Escherichia coli* (STEC) and the production of *stx*-phage particles and Shiga toxin. We tested the *in vitro* effect of lactic acid, acetic acid, citric acid, disodium phosphate, and sodium citrate on STEC growth. A bacteriostatic effect was observed in most of treated cultures. The exceptions were those treated with sodium citrate and disodium phosphate in which similar growth curves to the untreated control were observed, but with reduced OD_600_ values. Evaluation of phage production by plaque-based assays showed that cultures treated with sodium citrate and disodium phosphate released phages in similar o lower levels than untreated cultures. However, semi-quantification of Stx revealed higher levels of extracellular Stx in STEC cultures treated with 2.5% sodium citrate than in untreated cultures. Our results reinforce the importance to evaluate if additives and other treatments used to decrease bacterial contamination in food induce *stx*-phage and Stx production.

## Introduction

Shiga toxin-producing *Escherichia coli* (STEC) are important pathogens that can cause human diseases, like diarrhoea, haemorrhagic colitis, and haemolytic uraemic syndrome (HUS) (Karmali et al., 1985). STEC strains are characterized by their capacity to produce Shiga toxins, which are encoded by bacteriophages, usually named *stx*-phages. These phages influence the pathogenicity of STEC strains, since the expression of these toxins is upregulated when the lytic cycle of these phages is induced. They also play a role in the spread of *stx* among *E. coli* as well as to other bacteria (Schmidt et al., 1999; Muniesa et al., 2004; Probert et al., 2014). Several factors have been shown to induce the lytic pathway of *stx*-phages [revised by Krüger and Lucchesi (2015)]. Particularly, some antibiotics have been reported to increase phage induction and Stx production and therefore treatments of human STEC infections with some antibiotics may have adverse clinical consequences (Wong et al., 2000; Zhang et al., 2000; McGannon et al., 2010).

Meat consumption has been identified as one of the risk factors strongly associated with HUS (Bentancor et al., 2012) and several studies have shown that meat is frequently contaminated with STEC strains (Parma et al., 2000; Bosilevac and Koohmaraie, 2011). Food producing animals have been recognized as the most important source for the entry of STEC in the food chain (Arthur et al., 2002; Martin and Beutin, 2011), and ruminants, especially cattle, have been identified as the major reservoir of STEC strains (Caprioli et al., 2005; Mainil and Daube, 2005). In addition to meat and dairy products, other vehicles and transmission routes of STEC strains for human infection have been reported, like person-to-person contact and drinking and swimming water [reviewed by Doyle et al. (2006) and Kaspar et al. (2010)]. Several intervention strategies have been proposed to minimize meat contamination by STEC during slaughtering and meat processing, like hide washing (Arthur et al., 2007), hot water treatment (Bosilevac et al., 2006), high hydrostatic pressure (HHP) treatment (Gola et al., 2000), and treatments with organic acids and/or their salts (Eswaranandam et al., 2004; Over et al., 2009), among others.

Infectious *stx*-phages have been detected in minced beef and salad samples (Imamovic and Muniesa, 2011). Moreover, *stx*-encoding phages can be more resistant than their host bacteria to chlorination and heat treatments (Muniesa et al., 1999) and also have a high ability to tolerate exposure to several disinfectants (Rode et al., 2011). There is scarce information about the risk of *stx*-phage induction and *stx* gene dissemination to other bacteria in foods. Imamovic et al. (2009) showed that *stx* transduction in food matrices is possible under appropriate conditions. Studies are required to evaluate if antimicrobial interventions, as well as other farming practices and food processing technologies, may increase the rate of induction and propagation of *stx*-phages.

Several substances are used in the production, processing, treatment, packaging, transportation, and storage of food. In the meat industry, additives like citric acid and sodium citrate are widely applied for pH control, metal chelating, and preservation (Sammel et al., 2006). Phosphates are used in meat and meat products to adjust pH, sequester cations, change the ionic charges distributions, change the ionic strength, and/or to function as a bacteriostatic agent (Long et al., 2011). The aim of this study was to evaluate the *in vitro* effect of some additives on STEC growth, and on the production of *stx*-phage and Stx.

## Material and Methods

### Bacterial Strains

STEC strains FB3 (serotype O157:H7, *stx*_2_-positive) and FB5 (serotype O145:H-, *stx*_2_-positive) isolated from feedlot cattle (Padola et al., 2004), and the reference strain *E. coli* EDL933 (serotype O157:H7, positive for *stx*_1_ and *stx*_2_) were selected to evaluate the effect of additives on STEC growth. In addition, supernatants from *E. coli* EDL933 cultures were used to evaluate phage and Stx production. *E. coli* laboratory strain DH5α was used as host strain for *stx* phages in double-agar-layer plaque assays.

### Additives

The following compounds were prepared in stock solutions and used at indicated final concentrations: lactic acid (0.5 and 2.5% v/v), acetic acid (2.5% v/v), citric acid (0.5 and 2.5% w/v), disodium phosphate (0.1, 0.5, and 1% w/v), and sodium citrate (0.5, 1.0, and 2.5% w/v).

### Bacterial Growth/Lysis Curves

STEC strains were cultivated overnight in Luria Bertani (LB) medium at 37°C with shaking at 120 rpm. Aliquots (300 μl) were inoculated into 100 ml flasks containing 15 ml of fresh LB medium. The new cultures were incubated at 37°C and 120 rpm ~45 min. up to an optical density at 600 nm (OD_600_) ≈ 0.2-0.3 when each flask was added with the respective additive, or water (untreated control), or mitomycin C (final concentration of 0.5 μg/ml; positive control of phage induction), reaching a final culture volume of 15.5 ml. This moment was identified as 0 h of the assay. The incubation was continued at 37°C and 180 rpm for 18 h, and spectrophotometrically monitored every hour for the first 5 h. When it was necessary, the aliquots measured were previously diluted. In addition, viable bacterial count at 2 h was conducted by plating appropriate dilutions on LB agar plates. These assays were repeated at least two times.

### Evaluation of Phage Production

For phage quantification, aliquots of EDL933 cultures under different treatments were assayed. This strain was selected because phages induced from it showed lysis plaques easier to be counted by visual inspection than those from FB3 and FB5 strains. Aliquots from EDL933 cultures were taken at 3 h and centrifuged for 10 min at 10,000 × *g*, at 4°C. Supernatants were collected, filtered through low-protein-binding 0.22 μm membrane filters (Millex-GV, Millipore) and tenfold serially diluted for titration assays using the double-agar-layer method as follows. One hundred microliters of each dilution were mixed with 500 μl of an exponential phase culture of *E. coli* DH5α (OD_600_ ≈ 0.6–0.8) and incubated for 30 min at 37°C with shaking (120 rpm) (phage adsorption step). This suspension was then mixed with 3 ml of LB soft agar (0.75% w/v) supplemented with 9 mM CaCl_2_ and 1.5 μg/ml ampicillin and poured onto LB agar plates supplemented with 0.5–1 μg/ml ampicillin. After 18 h incubation at 37°C, lysis plaques were examined and enumerated. Ten lysis plaques were picked out from each plate and individually evaluated by PCR for *stx* carriage using a multiplex assay that detects *stx*_1_, *stx*_2_, and *eae* genes (Paton and Paton, 1998). This last gene was used to check the absence of chromosomal DNA from the STEC strain which could otherwise lead to false positive results in *stx*-phage PCR detection.

Phage quantification assays were repeated at least two times. To avoid possible interferences in the double-agar-layer method related with a possible chelating effect of disodium phosphate and sodium citrate, two more assays (named 3 and 4), were performed with addition of 100 μl CaCl_2_ 0.1 M in the phage adsorption step.

### Evaluation of Extracellular Shiga toxin

Stx production was semi-quantified by using an enzyme immunoassay (EIA, Ridascreen^®^ Verotoxin, R-Biopharm, Germany). Supernatants of cultures at 18 h were obtained by centrifugation at 12,000 ×*g* for 10 min, diluted 1/10 in LB and then analyzed according to manufacturer instructions. Regarding supernatants from cultures treated with acids, they were neutralized before performing the ELISA.

The results were spectrophotometrically measured at 450 nm and classified as weak positive (1+) if the extinction was >0.1–0.5 above the negative control, moderate (2+) (>0.5–1.0) and strongly positive 3+ (>1.0–2.0) to 4+ (>2.0). The assays were done twice.

## Results and Discussion

Some practices used along the food chain, like addition of substances, may influence bacterial growth and could increase the rate of induction and propagation of bacteriophages. This could represent a risk when the bacteriophages carry genes for potent toxins such as Stx. In this study, we evaluated the effect of different compounds used in the meat industry on the growth of STEC strains, and on the production of *stx*-phages and Stx.

Cultures of FB3, FB5, and EDL933 strains (control cultures and cultures exposed to different concentration of the compounds) were incubated and monitored spectrophoto metrically. For each treatment, similar OD_600_ patterns were observed for each of the three strains (results for EDL933 are shown). Cultures added with mitomycin C showed an increase in the OD_600_ during the first hours, reaching a maximum 2 h after induction, followed by a significant decrease, corresponding to a typical pattern of host cell lysis subsequent to induction of phage lytic cycle. This growth/lysis pattern was not observed in any of the cultures treated with the additives.

**FIGURE 1:**
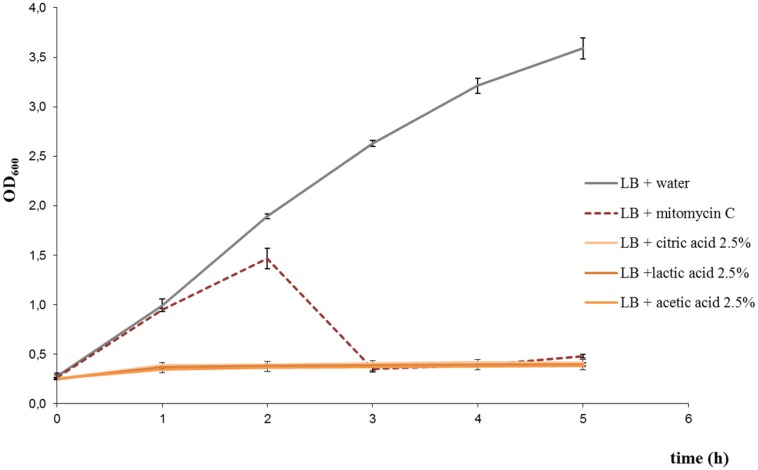
**Growth curves of *Escherichia coli* EDL933 strain under different treatments: 2.5% citric acid, 2.5% lactic acid, 2.5% acetic acid, and 0.5 μg/ml mitomycin C.** Growth curve for the untreated control is also shown. Similar results were obtained with *E. coli* FB5 and FB3 strains under the different treatments (not shown).

Cultures treated with lactic acid, acetic acid, or citric acid, at the concentrations tested, did not show an increase in OD_600_ over time (**Figure 1** shows the results for treatments with 2.5% citric acid, 2.5% lactic acid, and 2.5% acetic acid). Phage titration assays showed that phages in supernatants of cultures treated with acids were below the accepted range for countable phage plaques (around 3–5 plaques per plate could be observed from undiluted supernatants, which represented a ~2-log reduction in the plaque counts in comparison to the untreated control). In addition, extracellular Stx was not detected in the supernatants. Taking into account the results, those acids at tested concentrations inhibited the bacterial growth, without evidence of production of *stx*-phages and Stx.

**FIGURE 2:**
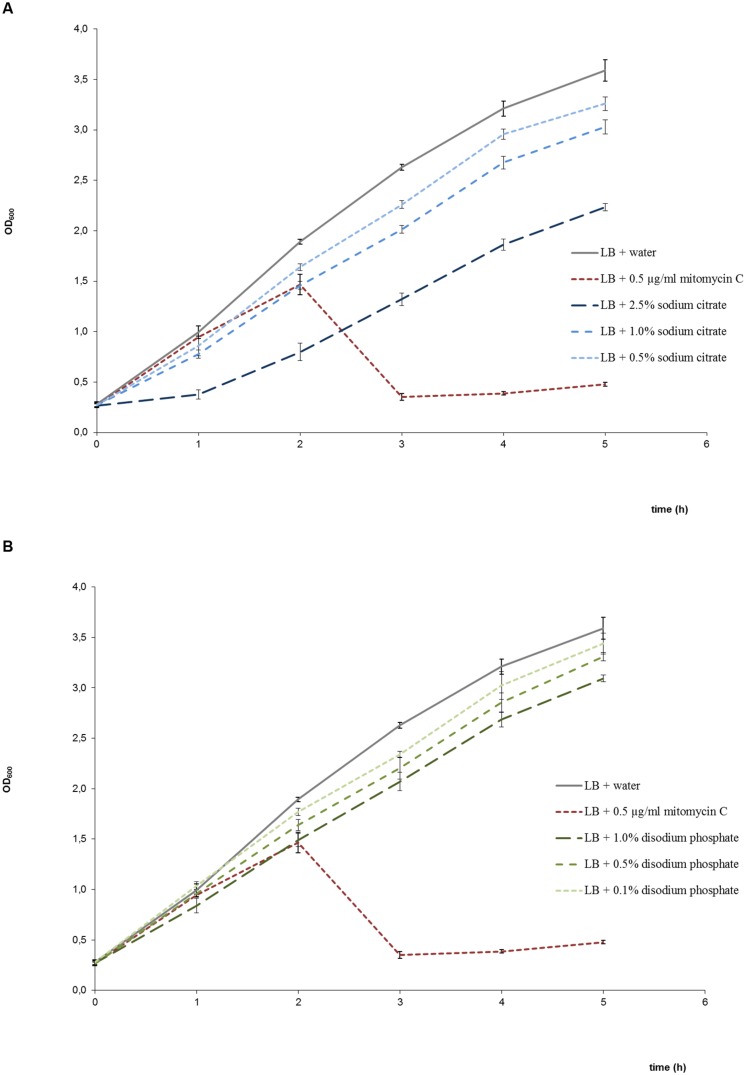
**Growth curves of *E. coli* EDL933 in LB medium in the presence and absence of sodium citrate **(A)**, and in the presence and absence of disodium phosphate (B).** Bacterial growth was determined by measuring the OD_600_. Each point corresponds to the average of four determinations ± standard error. Growth in the presence of mitomycin C was also included in both graphs as positive control of bacteriolysis due to phage lytic cycle induction.

**FIGURE 3:**
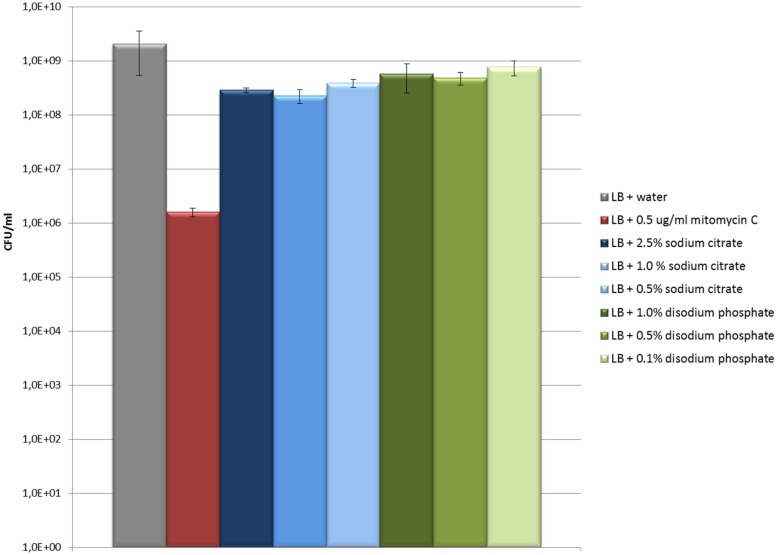
**Evaluation of the effect of sodium citrate and disodiun phosphate on *E. coli* EDL933 growth (CFU/ml).** The values and error bars on the graph are averages of three independent experiments.

Cultures treated with disodium phosphate or sodium citrate showed growth patterns similar to untreated cultures but with a reduction in the OD_600_ values with increasing salt concentrations (**Figures 2A,B**). Besides, viable bacterial counts showed that EDL933 cultures exposed to these salts had titers similar or slightly lower than untreated control cultures, and markedly higher than cultures with mitomycin C (**Figure 3**). In the first two phage titration assays, the supernatants of the cultures treated with disodium phosphate or sodium citrate contained phages below the accepted range for countable phage plaques, while titers of 3 × 10^2^ and 4 × 10^5^ plaque forming units (pfu)/ml were observed in untreated cultures and cultures with mitomycin C, respectively. The supplementation of the supernatants with CaCl_2_ in the adsorption step allowed quantification of phages from most of the cultures treated with salts, and also increased the number of pfu for both untreated and mitomycin C added controls. In all cases, the plaques analyzed by PCR were confirmed to correspond to *stx*_2_-phages.

**FIGURE 4:**
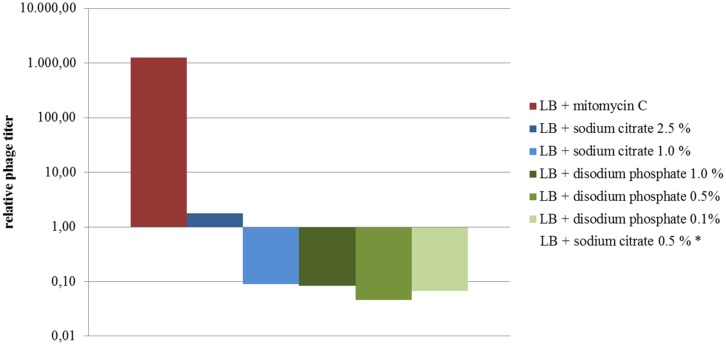
**Levels of phages expressed as the ratio of phage titers in supernatants of treated cultures relative to the untreated culture (results from assay 4 are shown).** The relative titers were plotted on a semilogarithmic scale. *Not shown (phage numbers below the accepted range for countable lysis plaques).

Phage titers observed for the supernatants of cultures treated with disodium phosphate and those with 0.5 and 1% sodium citrate were lower than those observed for untreated cultures (**Figure 4**). The exception was the culture treated with 2.5 % sodium citrate, which showed titers similar to the untreated control (**Figure 4**). Regarding extracellular Stx production, cultures treated with disodium phosphate presented similar results to the untreated control (**Table 1**). The cultures supplemented with 0.5% and 1.0% sodium citrate showed similar or slightly higher levels of Stx than the water control, respectively. Interestingly, cultures with 2.5% sodium citrate showed Stx levels considerable higher than those of untreated cultures.

**Table 1:** Semi-quantification of extracellular Shiga toxin by Ridascreen^®^ Verotoxin EIA (R-Biopharm, Germany).

Culture condition	Assay 1	Assay 4
LB + 2.5% sodium citrate	4+	4+
LB + 1.0% sodium citrate	2+	3+
LB + 0.5% sodium citrate	2+	2+
LB + 1.0% disodium phosphate	1+	2+
LB + 0.5% disodium phosphate	1+	2+
LB + 0.1% disodium phosphate	1+	1+
LB + 0.5 μg/ml mitomycin C	4+	4+
LB + water (untreated control)	1+	2+

*Supernatants of cultures at 18 h were analyzed after a 1/10 dilution in LB. Experiments were performed twice (assays 1 and 4).*

Altogether, the assays showed that sodium citrate and disodium phosphate at the tested concentrations slightly diminished the growth rate of the analyzed STEC strains without evidence of an increment of phage production. However, induction of *stx*-phage production cannot be ruled out, as phage can be produced but not released, or produced as non-infectious particles. Imamovic and Muniesa (2012) showed that chelation and the increase in *stx*_2_-phage induction are linked. In that study, culture treatment with 0.2 M sodium citrate showed effect on *stx*_2_ phage induction. Interestingly, our results showed an increase in Stx production in cultures treated with 2.5% sodium citrate in relation to the untreated control. The fact that production of Stx was increased in that condition could reinforce the previous arguments that *stx* phage production could be induced but not detected, or it could suggest Stx production independent of a complete phage production. It is important to note that EDL933 carries *stx*_1_ and *stx*_2_ genes, and both Stx1 and Stx2 toxin types can be detected by the Ridascreen kit. In consequence, we were not able to discriminate between Stx1 and Stx2 production, which are known to have differences in transcription control (Wagner and Waldor, 2002).

In the present study, we have shown that an additive, such as sodium citrate, although not having a strong effect on bacterial growth and phage production, can induce the production of Shiga toxin. Although we evaluated few STEC strains, and these results may not accurately represent the behavior of other strains, the present study alerts for a possible increase of Stx production by STEC in presence of some food additives.

Therefore, we consider that when testing the use of additives or other treatments applied in the food industry to decrease bacterial contamination, it is important to take into account the kind of bacteria that can be present. Particularly, in cases in which the bacteria can harbor phages encoding toxins that could be induced with the treatment.

## Author Contributions

LL, LM, JB: performed the experiments, participated in the acquisition, analysis and interpretation of the data, approved the final version of the paper. PL, AK: supervised the laboratory work, participated in the analysis and interpretation of the data, drafted the manuscript, and approved the final version of the paper.

## Conflict of Interest Statement

The authors declare that the research was conducted in the absence of any commercial or financial relationships that could be construed as a potential conflict of interest.
